# Spatial self-organization of confined bacterial suspensions

**DOI:** 10.1073/pnas.2503983122

**Published:** 2025-10-06

**Authors:** Babak Vajdi Hokmabad, Alejandro Martínez-Calvo, Sebastian Gonzalez La Corte, Sujit S. Datta

**Affiliations:** ^a^Department of Chemical and Biological Engineering, Princeton University, Princeton, NJ 08544; ^b^Princeton Center for Theoretical Science, Princeton University, Princeton, NJ 08544; ^c^Lewis-Sigler Institute for Integrative Genomics, Princeton University, Princeton, NJ 08540; ^d^Division of Chemistry and Chemical Engineering, California Institute of Technology, Pasadena, CA 91125

**Keywords:** bacteria, active matter, pattern formation, reaction–diffusion, self-organization

## Abstract

Lab studies of bacteria usually focus on cells in spatially extended, nutrient-replete settings, such as in liquid cultures and on agar surfaces. By contrast, many biological and environmental settings—ranging from mucus in the body to ocean sediments and the soil beneath our feet—feature multicellular bacterial populations that are confined to tight spots where essential metabolic substrates (e.g., oxygen) are scarce. What influence does such confinement have on a bacterial population? Here, we address this question by studying suspensions of motile *Escherichia coli* confined to quasi two-dimensional (2D) droplets. We find that when the droplet size and cell concentration are both large enough, the initially uniform suspension spatially self-organizes into a concentrated, immotile inner “core” that coexists with a more dilute, highly motile surrounding “shell.” By simultaneously measuring cell concentration, oxygen concentration, and motility-generated fluid flow, we show that this behavior arises from the interplay between oxygen transport through the droplet from its boundary, uptake by the cells, and corresponding changes in their motility in response to oxygen variations. Furthermore, we use biophysical theory and simulations to quantitatively describe this interplay. Our work thus sheds light on the rich collective behaviors that emerge for bacterial populations in confined environments, with implications for understanding ecological niches and engineering artificial systems.

## Significance

Bacteria often live in confined spaces (e.g., biological tissues, soil pores) where essential resources like oxygen are scarce. The population-scale implications of such confinement-induced chemical heterogeneities are poorly understood. Here, we show that when motile bacteria are confined to small droplets, they spontaneously organize in space—forming a concentrated immotile core surrounded by a shell of motile cells. This self-organization emerges from a feedback loop: cells consume oxygen, creating gradients that alter cellular motility and distribution, which in turn reshapes these gradients. We establish a biophysical model that quantitatively describes how this phenomenon depends on system parameters. Our findings could help understand bacterial ecological niches and inform approaches to engineer artificial active matter systems that self-organize through chemical feedback loops.

Bacteria often inhabit confined spaces, ranging from the thin mucus layer that coats the internal organs of most animals (1–7) to the pores in subsurface soils and sediments (8–14). In such environments, multicellular consumption of scarce metabolites can create strong gradients across a bacterial population (15–22). These metabolite gradients, in turn, can give rise to physiological heterogeneity throughout the population—even if its constituents are all genetically identical—since distinct cells experience different chemical microenvironments and thus behave differently (21–25).

This form of “phenotypic differentiation” can have profound implications for the organization and functioning of a population. For example, oxygen ($O_{2}$) consumption by aerobes can establish anoxic niches for obligate anaerobes, enabling both cell types to stably coexist (26–30)—facilitating the ability of such mixed populations to cycle nutrients in soil (28), perform key metabolic processes in the gut (31), and remove pollutants from dirty water (32). As a result, the influence of collectively generated metabolite gradients on confined bacterial populations is well studied in the context of variations in gene expression, metabolic activity, chemical signaling, and growth/proliferation (11, 16, 33–42).

Less studied, however, is how such gradients shape, and are shaped by, cellular motility—another fundamental characteristic of many bacteria. Studies of unconfined populations of motile cells have revealed that they migrate as coherent fronts in response to self-generated gradients of chemoattractants/$O_{2}$, a phenomenon known as collective chemotaxis/aerotaxis (43–59). And remarkably, cells of different motility characteristics self-sort within such a front in response to the shape of the gradient (44, 60). The implications of self-generated metabolite gradients on confined populations of motile bacteria are, however, less explored.

Here, we report a distinct form of phenotypic differentiation that arises in confined populations of motile aerobic bacteria. We study initially uniform suspensions of swimming *E. coli* confined to quasi-2D droplets. When the droplet is sufficiently large and concentrated, we find that the population spontaneously self-organizes into a concentrated inner core of immotile cells surrounded by a more dilute annular shell of highly motile cells—even though all the cells are genetically identical. By integrating experiments, theory, and simulations, we show that this phenomenon is governed by the interplay between cellular aerobic respiration, $O_{2}$ transport and availability, and cellular motility. In particular, $O_{2}$ supplied from the droplet boundary is taken up by the swimming cells, eventually becoming depleted and causing cells in the core to rapidly lose motility—as has been studied in other contexts (29, 61–63). In some cases, this spatial self-organization is permanent. In others, the population continues to restructure itself over longer time scales: Aerotaxis enables the swimming cells to reshape the $O_{2}$ gradient in turn and promote $O_{2}$ influx, causing the core to eventually disappear. We establish quantitative principles that describe the conditions under which anoxic core formation arises and whether it is transient or permanent—in excellent agreement with all our experiments and simulations. Hence, this work sheds light on the fascinating behaviors that can emerge from the interplay of confinement, collectively generated metabolite gradients, and cellular motility. In doing so, it provides principles for predicting and controlling the spatial organization of bacterial populations, and other forms of chemically reactive living and active matter, more broadly.

## Results

### Confined E. coli Suspensions Spontaneously Self-Organize in Three Distinct Ways.

**Fig. 1.**
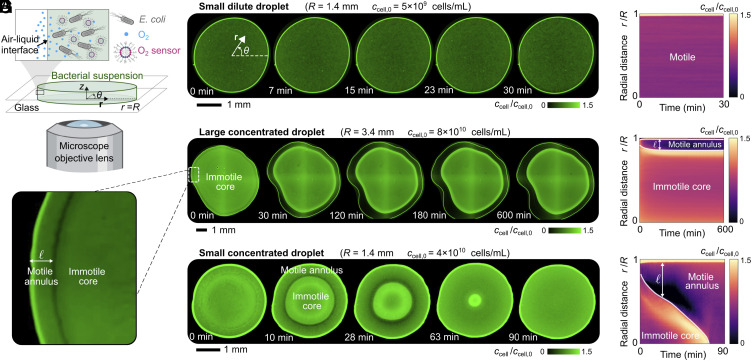
Spontaneous spatial self-organization of confined bacterial suspensions. (*A*) Schematic of the experimental setup. A droplet containing swimming fluorescent *E. coli* (gray) is sandwiched between two flat glass plates separated by a gas-permeable spacer and maintained in a humid environment at 30 °C. Oxygen (blue) diffuses in from the air–liquid interface and is detected using a dissolved fluorescent probe encapsulated in phospholipid micelles (pink). Depth-averaged bright-field micrographs showing the time evolution of bacterial suspensions (Movies S1–S4) are shown for (*B*) and (*C*) a small dilute droplet, *D* and *E* a large concentrated droplet, and *F* and *G* a small concentrated droplet, corresponding to $\left(\tilde{R},{\tilde{k}}_{t}^{-1}\right)=\left(0.96,2.8\right)$, (9.3,0.7), and (2.7,1), respectively. The kymographs in (*C*, *E*, and *G*) represent these dynamics by showing the azimuthally averaged cell concentration $c_{\mathrm{cell}}$, normalized by its initial spatially uniform value $c_{\mathrm{cell}},0$, as a function of radial position r and time t. The small dilute droplet remains nearly uniform; by contrast, in more concentrated droplets a concentrated core of immotile cells forms, surrounded by a less-concentrated annular shell of motile cells. In large droplets, this core forms permanently, while in small droplets, this core shrinks and eventually disappears. As shown in *SI Appendix*, we do not detect measurable z variations in the motile annulus.

To explore the collective dynamics of confined motile bacteria, we sandwich droplets of suspensions of swimming *E. coli* between two flat glass plates (Fig. 1*A*) in a humid environment to minimize evaporation. The gap between the plates is 130μm high, much smaller than the droplet radius R, which we set to be between 0.3 and 3.5 mm. The plates themselves are $O_{2}$-impermeable, and so, $O_{2}$ can only enter from the air–liquid interface at the droplet perimeter. To avoid confounding effects, we use a buffer that only contains nonmetabolized salts; hence, the cells do not proliferate, but instead, use their internal resources to maintain motility over many hours and only respond to variations in $O_{2}$ [*SI Appendix*, Fig. S1, (62)]. The cells are initially uniformly dispersed at a concentration $c_{\text{cell},0}$. They constitutively express green fluorescent protein, enabling us to map subsequent variations in the depth (z)-integrated cellular concentration $c_{\mathrm{cell}}\left(r,t\right)$ throughout each droplet via confocal microscopy using both bright-field and fluorescence imaging; r and t represent the radial coordinate and time, respectively.

We start with a small (R=1.4mm) and dilute ($c_{\mathrm{cell},0}=5\times 10^{9}\;$cells/mL) droplet. In this case, the entire population remains motile for the duration of the experiment, as shown in Movie S1. Almost all the cells are uniformly dispersed, with a minute fraction localized at the air–liquid interface, as shown by the bright thin band along the droplet perimeter in Fig. 1 *B* and *C*.

We observe strikingly different behavior in the case of a larger (R=3.4mm) and more concentrated ($c_{\mathrm{cell},0}=8\times 10^{10}\;$cells/mL) droplet. Instead of being uniformly dispersed, the cells rapidly self-organize into two distinct domains (*SI Appendix* and Movie S2): a large, concentrated inner core surrounded by a thinner, more dilute annular shell, as shown by the brighter and darker regions, respectively, in Fig. 1*D*. A minute fraction of cells again localizes at the air–liquid interface. The cells in the different domains have markedly different motility characteristics; in the core, cells are completely immotile, whereas in the annulus, they remain fully motile, as shown in Movie S3. Interestingly, the core shrinks slightly, and cells accumulate near its periphery and are depleted from the surrounding annulus, during the first ∼100 min. The core then reaches a steady-state size, as shown in Fig. 1*E*. Correspondingly, the surrounding annulus has a nearly constant width ℓ∼300μm, following the shape of the droplet boundary and even tracking slight imperfections in droplet shape (Fig. 1*D*).

We observe yet another different behavior in the case of a small (R=1.4mm) but still concentrated ($c_{\mathrm{cell},0}=4\times 10^{10}\;$cells/mL) droplet. The cells again rapidly self-organize into a concentrated inner core, in which they are immotile, surrounded by a more dilute annular shell in which they remain motile (Movie S4). Moreover, cells in the core again appear to accumulate near its periphery and are depleted from the surrounding annulus. However, we observe two key differences from the previous case. First, a larger fraction of the motile cells localizes at the air–liquid interface, as shown by the thicker bright band along the droplet perimeter in Fig. 1*F*. Second, the self-organization into a core–shell structure is transient. As time progresses, the core appears to be “eroded” by the shell, progressively shrinking until it ultimately disappears after ∼70 min (Fig. 1 *F* and *G*), reaching a final steady state similar to the first case shown in Fig. 1 *B* and *C*.

### Bacterial Self-Organization Is Governed by the Interplay Between Oxygen Transport and Availability, Cellular Respiration, and Motility.

Why do confined, concentrated bacterial populations self-organize into a core–shell structure? And why is this spatial organization permanent in some cases and transient in others? Close inspection of the cellular swimming patterns in concentrated droplets (Movie S3), obtained using high-magnification imaging 30μm above the bottom glass plate, provides a clue. Just outside the core, the time-averaged velocity field associated with bacterial swimming splits into two coherent, radially directed streams (*SI Appendix*, Figs. S2 and S3). The stream closer to the core is directed radially inward (pink in *SI Appendix*, Fig. S2), indicating motile cells that swim inward and rapidly lose motility, causing them to accumulate at the core periphery—that is, the core acts as a *sink* for cells. The outer stream is directed radially outward (green in *SI Appendix*, Fig. S2), resulting in eventual depletion of cells from the region surrounding the core and localization of cells at the air–liquid interface instead. These rapid changes in motility suggest that a metabolic shift is at play—presumably in response to spatial variations in dissolved $O_{2}$, given that it is the only exogenous metabolite available to the cells in our experiments, and the cells use it to perform aerobic respiration. Indeed, prior studies (61, 62) have shown that *E. coli* rapidly loses motility, just as we observe in the core, when deprived of $O_{2}$. Other studies have shown that when $O_{2}$ is available, *E. coli* biases its swimming toward regions of higher $O_{2}$ availability (45), just as we observe near the air–liquid interface. Moreover, large, concentrated droplets that are not confined by an overlying glass plate as in our experiments, but are instead open to atmospheric $O_{2}$, do not exhibit core formation (64). Taken altogether, these observations suggest that the self-organization of confined *E. coli* suspensions is governed by the interplay between oxygen transport and availability, cellular respiration, and motility.

**Fig. 2.**
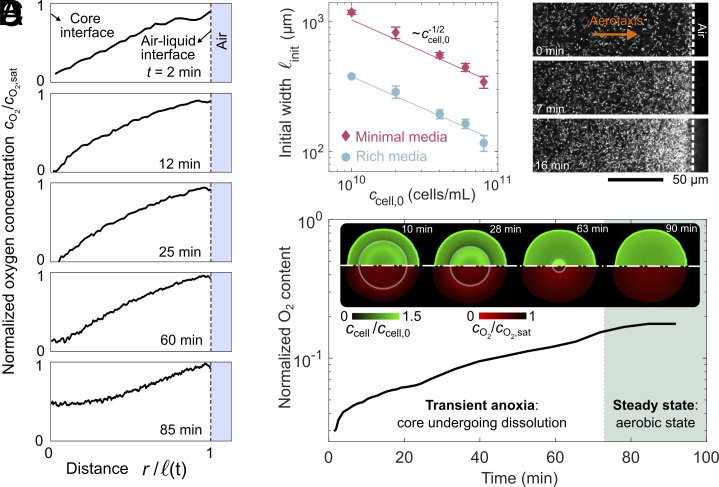
Bacteria regulate oxygen transport through uptake and by accumulating at the air–liquid interface. (*A*) Evolution of the azimuthally averaged profile of oxygen concentration $c_{{\text{O}}_{2}}$, normalized by its saturation concentration $c_{{\text{O}}_{2},\text{sat}}$, as a function of radial position across the annular shell, r; ℓ is the time (t)-dependent annulus width. This measurement is for a droplet with R=1.4 mm and $c_{\text{cell},0}=4\times 10^{10}\mathrm{cells}/\mathrm{mL}$, corresponding to $\tilde{R}=2.7$ and ${\tilde{k}}_{t}^{-1}=1$. Cellular uptake creates an ${\text{O}}_{2}$ gradient, with $c_{{\text{O}}_{2}}\approx c_{{\text{O}}_{2},\text{sat}}$ at the air–liquid interface and $c_{{\text{O}}_{2}}\approx 0$ at the periphery of the core. Over time, however, influx of ${\text{O}}_{2}$ causes it to reaerate the core. (*B*) Variation of the initial width of the motile annulus $\ell_{\mathrm{init}}$ after core formation with increasing cell concentration $c_{\text{cell},0}$ in minimal media (BMB, red) and in nutrient-rich media (LB Broth, blue). The lines show excellent agreement with our theoretical prediction for the oxygen penetration length $l_{{\text{O}}_{2}}$, with uptake rates $k_{O_{2}}=4\times 10^{4}\;\text{molecules}\;{\text{s}}^{-1}{\text{cell}}^{-1}$ in minimal media and $3\times 10^{5}\;\text{molecules}\;{\text{s}}^{-1}{\text{cell}}^{-1}$ in nutrient-rich media, consistent with prior measurements (44, 61, 65). (*C*) High magnification fluorescent confocal micrographs taken at a planar optical section in the middle of the channel (Movie S6) reveal cell accumulation near the air–liquid interface with time due to aerotaxis. Experiment was done at R=1.4 mm and $c_{\text{cell},0}=10^{10}\mathrm{cells}/\mathrm{mL}$, corresponding to $\tilde{R}=0.43$ and ${\tilde{k}}_{t}^{-1}=0.16$. (*D*) Time evolution of the total ${\text{O}}_{2}$ content of the droplet in *A* obtained by integrating the measured ${\text{O}}_{2}$ concentration over the annulus, normalized by the total ${\text{O}}_{2}$ content at the saturation condition. *Inset* shows micrographs of the profiles of cells (top half) and dissolved ${\text{O}}_{2}$ (bottom half) throughout the droplet (*SI Appendix*, Movie S5). The bacteria are imaged via depth-averaged bright-field microscopy and the dissolved ${\text{O}}_{2}$ are imaged using fluorescent confocal microscopy from an optical slice that is ∼100μm thick. Note that the dimming of the ${\text{O}}_{2}$ signal within the anoxic core area is due to light scattering from the concentrated layer of bacteria sedimented on the bottom surface. Our measurements show that after the core forms, enhanced ${\text{O}}_{2}$ influx is concomitant with core shrinkage (“transient anoxia”), ultimately causing it to disappear and marking the final aerobic steady state.

To test this idea, we use a fluorescent probe (61) to directly map variations in dissolved $O_{2}$ throughout a confined droplet (Movie S5). The droplet is small and concentrated, and so, exhibits transient core–shell formation as in Fig. 1*F*. Immediately after the start of the experiment, we observe a radial gradient in $O_{2}$ concentration, $c_{{\text{O}}_{2}}$, progressively decreasing from its saturation value $c_{{\text{O}}_{2},\text{sat}}$ at the outer air–liquid interface inward and eventually reaching zero at the periphery of the core (Fig. 2*A*). That is, the formation of a core of immotile cells is indeed coincident with the establishment of an anoxic region inside the droplet—which arises presumably because of uptake by the swimming cells in the surrounding annular shell. If this conjecture is correct, then the annulus width ℓ should be set by the distance over which ${\text{O}}_{2}$ supplied from the droplet boundary can diffuse radially inward before being fully consumed by the swimming cells: $l_{{\text{O}}_{2}}\approx \sqrt{D_{{\text{O}}_{2}}c_{{\text{O}}_{2},\text{sat}}/\left(k_{O_{2}}c_{\text{cell,0}}\right)}$ (65), where $D_{{\text{O}}_{2}}$ is the diffusivity of ${\text{O}}_{2}$ and $k_{O_{2}}$ is the maximal ${\text{O}}_{2}$ uptake rate per cell. We directly test this prediction by varying $c_{\text{cell},0}$ over an order of magnitude and measuring the corresponding ℓ. As shown by the red points in Fig. 2*B*, our experimental measurements confirm this prediction. As an additional test, we perform the same experiments, but for cells dispersed in a nutrient-rich liquid—in which active metabolism of nutrients increases the rate of ${\text{O}}_{2}$ uptake, $k_{O_{2}}$. Our measurements again confirm this prediction, as indicated by the blue points in Fig. 2*B*. These data quantitatively establish that, in large and concentrated droplets, ${\text{O}}_{2}$ depletion due to uptake by swimming cells in the outer annular shell causes inner cells to lose motility, sediment, and form a concentrated core.

Having identified the mechanism of core formation, we next ask: why, in some cases, is the core eventually eroded away? Examining the ${\text{O}}_{2}$ and cell concentration profiles simultaneously, which shows how they are coupled, helps to address this question. Not only does ${\text{O}}_{2}$ uptake cause the core of the droplet to become anoxic, but it also establishes a radial gradient through the annular shell (Fig. 2*A*) that the swimming cells respond to via aerotaxis. An example is shown in Fig. 2*C* (Movie S6). As these swimming cells accumulate near the air–liquid interface, their respiration continues to reshape this ${\text{O}}_{2}$ gradient, promoting its further influx from the droplet periphery. Our $c_{{\text{O}}_{2}}$ measurements directly confirm this picture. We integrate them both radially (r) and azimuthally (θ) to determine the total amount of ${\text{O}}_{2}$ in the droplet: $\int_{0}^{2\pi }\int_{R-\ell }^{R}c_{{\text{O}}_{2}}\left(r,\theta \right)r\;dr\;d\theta$. As shown in Fig. 2*D*, ${\text{O}}_{2}$ content monotonically increases over time, concurrent with the erosion of the anoxic core. This process persists until the core disappears altogether (Fig. 2*D*, *Inset*), resulting in a final aerobic steady state. By biasing its distribution within the outer annulus via aerotaxis, the motile subpopulation of cells promotes ${\text{O}}_{2}$ influx across the air–liquid interface; as a result, core formation is only transient.

### Quantitative Principles Underlying Bacterial Self-Organization.

**Fig. 3.**
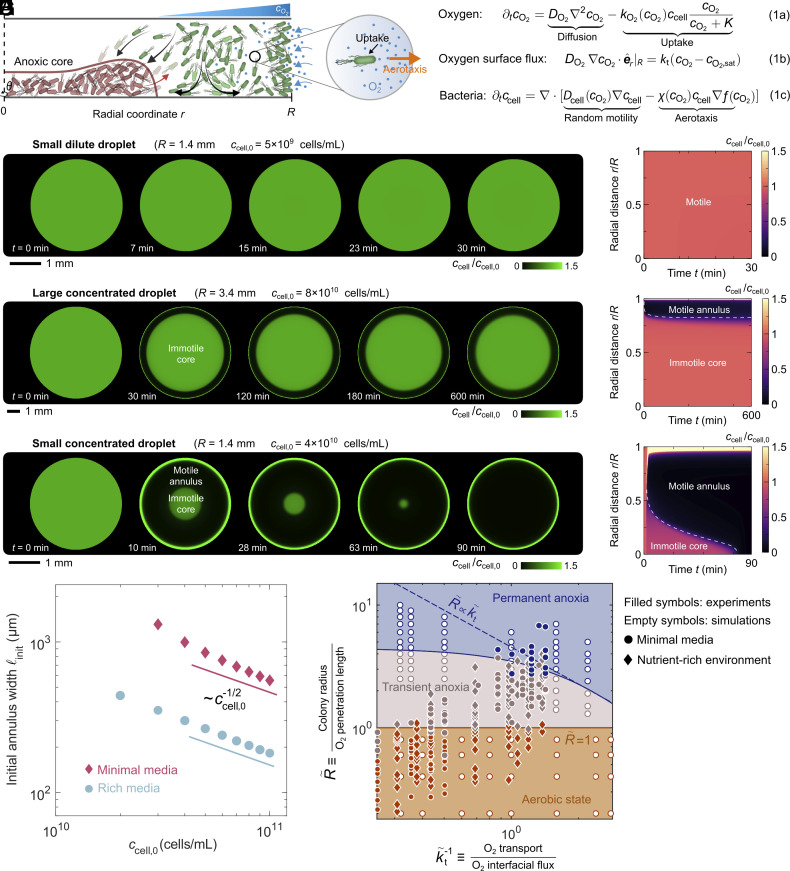
Theoretical model recapitulates the experimental observations and unifies them in a morphological state diagram. (*A*) Schematic of the biophysical principles underlying core formation and shrinkage. Motile bacteria in the annulus (green) swim either randomly into the anoxic core (left-pointing arrows), where they lose motility (red), settle, and concentrate themselves, or they move up the ${\text{O}}_{2}$ gradient via aerotaxis (right-pointing arrow) and accumulate at the air–liquid interface. They do not block the influx of ${\text{O}}_{2}$, which is much smaller (71) than the interstices between cells. Conversely, their uptake promotes further ${\text{O}}_{2}$ influx, which can cause cells at the core periphery to become motile again (right-pointing red arrow), driving core erosion and shrinkage. These feedbacks between ${\text{O}}_{2}$ transport and influx, its uptake by cells, and cellular motility are quantified by Eqs. **1a–1c**, along with the condition that at the air–liquid interface, there is no flux of bacteria from the liquid phase into the gas i.e., $\left[-D_{\text{cell}}\nabla c_{\text{cell}}+\chi c_{\text{cell}}\nabla f\left(c_{{\text{O}}_{2}}\right)\right]\cdot {\hat{e}}_{r}{|}_{r=R}=0$. To describe the loss of bacterial motility at low O_2_ concentration, we use the phenomenological expressions $D_{\text{cell}}\left(c_{{\text{O}}_{2}}\right)=\frac{D_{\text{cell},0}}{2}\left(1+\tanh\left[\left(c_{{\text{O}}_{2}}-c_{\text{crit}}\right)/\delta \right]\right)$, $\chi \left(c_{{\text{O}}_{2}}\right)=\frac{\chi_{0}}{2}\left(1+\tanh\left[\left(c_{{\text{O}}_{2}}-c_{\text{crit}}\right)/\delta \right]\right)$, and $k_{{\text{O}}_{2}}\left(c_{{\text{O}}_{2}}\right)=\frac{k_{{\text{O}}_{2},0}}{2}\left(1+\tanh\left[\left(c_{{\text{O}}_{2}}-c_{\text{crit}}\right)/\delta \right]\right)$, where δ controls the sharpness of the transition at $c_{{\text{O}}_{2}}=c_{\text{crit}}$. (*B*–*G*) Numerical simulations of the time evolution of bacterial suspensions (Movies S7–S9) for the exact same conditions as the experiments in Fig. 1*B*–*G*, using experimentally measured values for the parameters as described in the *SI Appendix*. We solve the model in the radial domain assuming that all fields are uniform along both the azimuthal angle (axisymmetry) and the vertical direction z, reducing it to a one-dimensional system of two equations evolving over time. As in the experiments, the small dilute droplet is uniform, while more concentrated droplets have an inner concentrated core of immotile cells surrounded by a less-concentrated annular shell of motile cells that is either permanent or transient. (*H*) The simulations also recapitulate the experimental measurements of the initial annulus width shown in Fig. 1*H*. (*I*) State diagram is spanned by the two dimensionless parameters $\tilde{R}$ and ${\tilde{k}}_{t}^{-1}$, where the former compares the droplet radius to the ${\text{O}}_{2}$ penetration length, and the latter compares the rates of ${\text{O}}_{2}$ transport via diffusion and influx from the air–liquid interface. Filled and empty symbols show experimental and simulation results, respectively. Circles and diamonds indicate minimal media and nutrient-rich conditions, respectively. The solid dark orange line shows our theoretical prediction for the boundary between the aerobic state and transient anoxia, $\tilde{R}=1$. The dashed dark blue line shows our theoretical prediction for the boundary between the transient and permanent states of anoxia, $\tilde{R}\propto {\tilde{k}}_{t}$, which assumes that ${\text{O}}_{2}$ is not saturated at the air–liquid interface, appropriate for ${\tilde{k}}_{t}^{-1}>1$; the solid dark blue line shows this boundary without making this assumption, as determined by simulations. In all cases, we find excellent agreement between theory, simulations, and experiments.

To further rationalize the experimental observations, we construct a continuum model that describes the interplay between oxygen transport and availability, cellular respiration, and motility in confined bacterial suspensions. Given that the experimental droplets are quasi-2D, for simplicity, we consider depth (z)-integrated quantities in an axisymmetric 2D system. The model is summarized in Fig. 3*A*. Eq. **1a** relates local changes in ${\text{O}}_{2}$ levels to its diffusion and uptake by the motile cells, which in turn is modulated by oxygen availability relative to the characteristic concentration K=1μM via Michaelis–Menten kinetics. Eq. **1b** provides a boundary condition relating ${\text{O}}_{2}$ influx at the air–liquid interface, $D_{{\text{O}}_{2}}\nabla c_{{\text{O}}_{2}}\cdot {\hat{e}}_{r}{|}_{r=R}$, to the difference between $c_{{\text{O}}_{2}}$ and $c_{{\text{O}}_{2},\text{sat}}$ through the mass transfer coefficient $k_{t}$; here, ${\hat{e}}_{r}$ is the unit radial vector. Finally, Eq. **1c** relates local changes in bacterial concentration to their motility, which has two components—undirected random motion and biased random motion up an ${\text{O}}_{2}$ gradient. The former can be described as a diffusive process with an “active” diffusivity $D_{\text{cell}}$ (66). The latter is caused by spatial variations in the rate at which cells reorient (67), which when coarse-grained to the population scale yields the aerotactic velocity $\chi \nabla f(c_{{\text{O}}_{2}})$ (44, 57–59, 68, 69). Here, the monotonically increasing function $f\left(c_{{\text{O}}_{2}}\right)=c_{{\text{O}}_{2}}/\left(K_{\chi }+c_{{\text{O}}_{2}}\right)$, validated directly in experiments (46), describes the ability of the cells to sense variations in ${\text{O}}_{2}$ based on first-order kinetics of receptor binding with a dissociation constant $K_{\chi }=7\;\mu$M, and the aerotactic coefficient χ describes their ability to move up the sensed gradient. Multiplying the aerotactic velocity by $c_{\text{cell}}$ therefore describes the aerotactic bacterial flux. Importantly, to account for the loss of cellular motility when deprived of $O_{2}$, both motility parameters $D_{\text{cell}}$ and χ become negligible when $c_{{\text{O}}_{2}}$ drops below a critical value $c_{\text{crit}}\ll K_{\chi }$ (61). For simplicity, we do not incorporate any other changes in cellular physiology that may arise. Given that *E. coli* can adapt to starvation and survive for days with minimal lysis (70), we also assume that $O_{2}$-deprived cells do not lyse and release nutrients for the other bacteria; indeed, comparing the integrated light intensity—which represents the total cell count in the drop—at the beginning of our experiments to that after the core disappears indicates that the total cell count varies by less than 1%.

Remarkably, numerical simulations of this minimal model—using measured values for all parameters, either from our experiments or prior literature (detailed in *SI Appendix*)—show that it recapitulates all the key features of the experimental observations. When the droplet is small and dilute, it remains fully aerobic; therefore, the entire population remains motile and nearly uniformly dispersed, as shown in Fig. 3 *B* and *C* and Movie S7 (compare to Fig. 1 *B* and *C* and Movie S1). When the droplet is larger and more concentrated, the cells self-organize into a concentrated immotile core—which remains permanently anoxic—surrounded by a dilute motile annular shell of width ℓ∼300μm, as shown in Fig. 3 *D* and *E* and Movie S8 (compare to Fig. 1 *D* and *E* and Movie S2). Moreover, we find the same dependence of this annular shell width on $c_{\text{cell},0}$ as in the experiments, as shown in Fig. 3*H* (compare to Fig. 2*B*). Finally, when the droplet is small but concentrated, a larger fraction of motile cells localizes at the air–liquid interface, and anoxic core formation is transient, just as in the experiments, as shown in Fig. 3 *F* and *G* and Movie S9 (compare to Fig. 1 *F* and *G* and Movie S4). These results indicate that the biophysical picture described in Fig. 3*A* captures the essential processes underlying the spatial self-organization of confined bacterial suspensions.

Analysis of the model also enables us to establish quantitative principles describing the conditions under which each of the three states observed—aerobic, permanently anoxic core–shell, and transiently anoxic core–shell—arises. To do so, we first nondimensionalize the governing equations in Fig. 3*A*, revealing two dimensionless parameters that describe a confined bacterial suspension (*SI Appendix*): $\tilde{R}\equiv R/l_{{\text{O}}_{2}}$, which compares the size of the droplet to the distance ${\text{O}}_{2}$ penetrates into it before being fully consumed, and ${\tilde{k}}_{t}^{-1}\equiv D_{{\text{O}}_{2}}/\left(k_{\text{t}}l_{{\text{O}}_{2}}\right)$, which compares the rates of ${\text{O}}_{2}$ transport via diffusion, $∼D_{{\text{O}}_{2}}/l_{{\text{O}}_{2}}^{2}$, and influx from the air–liquid interface, $∼k_{t}/l_{{\text{O}}_{2}}$. The conditions demarcating transitions between the three states of a confined bacterial suspension can then be expressed in terms of these two parameters. Indeed, a necessary condition for an anoxic core to form, whether transiently or permanently, is that $\tilde{R}>1$; otherwise, ${\text{O}}_{2}$ can freely penetrate throughout the droplet, and the aerobic, core-free state is maintained. Furthermore, when $\tilde{R}>1$, we expect that anoxic core formation is permanent when ${\text{O}}_{2}$ uptake by the entire population, $∼k_{O_{2}}c_{\text{cell},0}R$, outpaces influx from the air–liquid interface, which we estimate as $∼k_{t}c_{O_{2},\text{sat}}$ when ${\text{O}}_{2}$ influx is not limited by saturation at the air–liquid interface. That is, we expect that for ${\tilde{k}}_{t}^{-1}>1$, anoxic core formation is permanent when $\tilde{R}>{\tilde{k}}_{t}$.

To test these predictions quantitatively, we perform 256 different experiments and 97 different simulations, systematically exploring a broad range of droplet radii R and cell concentrations $c_{\text{cell},0}$ in both minimal and nutrient-rich liquid media. The results are summarized by the points in Fig. 3*I*, which represents a morphological state diagram spanned by the two dimensionless control parameters revealed by our analysis, $\tilde{R}$ and ${\tilde{k}}_{t}^{-1}$. The three different colors represent the three different states observed: dark orange represents conditions under which the droplet remains in the aerobic, fully motile state, while gray-orange and blue represent conditions under which the droplet self-organizes into an anoxic, immotile core surrounded by an aerobic, motile shell either transiently or permanently, respectively. Points in these three different states cluster in different regions of the diagram, confirming the utility of $\tilde{R}$ and ${\tilde{k}}_{t}^{-1}$ as dimensionless control parameters. The transition from the aerobic state to that of anoxic core formation is described well by the predicted $\tilde{R}=1$, shown by the horizontal dark orange line. Moreover, when ${\tilde{k}}_{t}^{-1}>1$, the transition from transient to permanent anoxic core formation is also described well by the predicted $\tilde{R}\propto {\tilde{k}}_{t}$ with a prefactor ≈5 obtained by fitting to the boundary between the simulation results for permanent and transient anoxia at the largest values of ${\tilde{k}}_{\text{t}}^{-1}$, as shown by the dashed blue line. Conversely, for ${\tilde{k}}_{t}^{-1}<1$, ${\text{O}}_{2}$ reaches its saturation concentration at the air–liquid interface (*SI Appendix*), constraining its influx and removing the dependence of the transition from transient to permanent anoxic core formation on ${\tilde{k}}_{t}^{-1}$—as expected, and as quantitatively shown by the solid blue line $\tilde{R}=18.9/\left(4.1+1.9{\tilde{k}}_{\text{t}}^{-1}\right)$, which provides a reasonable fit to both experimental and numerical results in both limits. Thus, our integrated experiments, theoretical analysis, and simulations establish biophysical principles that quantitatively describe how confined bacterial suspensions self-organize across different levels of confinement.

### Viscoelasticity Generates Strong Fluctuations During Core Erosion.

In many cases, bacteria are confined to polymeric fluids, such as mucus in the body (72–75), exopolymers in the ocean, and extracellular polymeric matrices in biofilms (76). Recent work has shown that the viscoelasticity of these fluids can alter how individual motile cells swim in unusual ways—for example, by markedly increasing the swimming speed (77–83) and enhancing fluid-mediated interactions between cells (64, 84, 85). Thus, as a final exploration of bacterial self-organization in confinement, we ask: how do interactions with extracellular polymers influence this process?

To address this question, we repeat the experiment shown in Fig. 1*F*, but with 5 MDa polyethylene oxide (PEO) added to the fluid at a semidilute concentration (0.2wt.%) comparable to that of mucus in the body. This synthetic polymer is chemically inert, uncharged, nonadsorbing, and similar in size to many biological mucins. Our measurements confirm that cell swimming is enhanced in this polymeric fluid, with an approximately fourfold increase in $D_{\mathrm{cell}}$ compared to the polymer-free case (*SI Appendix*, Table S1). The experiment is characterized by $\tilde{R}=2.7$ and ${\tilde{k}}_{t}^{-1}=1$, and so, we expect that just like the polymer-free case, an anoxic immotile core forms, surrounded by an oxygenated motile annular shell, and then disappears (Fig. 3*I*). However, simulation indicates that because of their enhanced motility, cells accumulate at the air–liquid interface more rapidly than in the polymer-free case, promoting ${\text{O}}_{2}$ influx and causing the core to shrink faster (compare Movies S4 and S10).

**Fig. 4.**
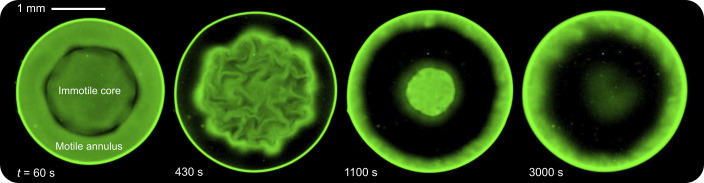
Fluid viscoelasticity imparts strong and coherent fluctuations during bacterial self-organization. Depth-averaged bright-field microscopy micrographs showing the time evolution of a bacterial suspension (Movie S11) with R=1.4 mm and $c_{\text{cell},0}=4\times 10^{10}\mathrm{cells}/\mathrm{mL}$, corresponding to $\tilde{R}=2.7$ and ${\tilde{k}}_{t}^{-1}=1$, in a viscoelastic solution of 5 MDa PEO at 0.2wt.% (≈4 times its overlap concentration). As our theory predicts, an immotile core forms, surrounded by a motile annulus. However, the swimming cells in the annulus generate coherent swirls that mix the core. More cells also localize at the air–liquid interface, causing the core to shrink faster than the polymer-free case.

Our experimental observations confirm these predictions, but also reveal additional rich dynamics imparted by fluid viscoelasticity. As shown in Movie S11 and Fig. 4, a concentrated immotile core initially forms (t≲300 s), with cells accumulating near its periphery and depleting from the surrounding annulus. However, unlike the polymer-free case, we observe large (∼100 μm) coherent swirls generated by the enhanced cellular swimming (63, 86) at the periphery of the core. As time progresses (t≳300 s), these swirls propagate into and throughout the core. Concomitantly, a large fraction of motile cells—much larger than in the polymer-free case, as seen by comparing the third panels of Figs. 1*F* and 4—localizes at the air–liquid interface. As before, the increased ${\text{O}}_{2}$ influx then causes the core to continue to shrink until it has eventually disappeared (t≳2,900 s)—faster than in the polymer-free case of Fig. 1*F*. Additionally, unlike the polymer-free case, the cells are not uniformly distributed throughout the droplet at the end of the experiment; rather, they are depleted from the interior and instead remain localized at the air–liquid interface, as shown by the thick band in the last panel of Fig. 4. Taken altogether, these observations demonstrate that our central finding—that confined bacterial suspensions self-organize into core–shell structures due to ${\text{O}}_{2}$ limitation—is more general, manifesting in complex fluids akin to those encountered in many biological settings. Further unraveling the additional dynamics imparted by fluid viscoelasticity will be a fascinating direction for future work.

## Discussion

Our work elucidates how bacterial suspensions spatially self-organize into concentric core–shell structures when confined to droplets, driven by the interplay between ${\text{O}}_{2}$ transport, cellular metabolism, and motility. By simultaneously measuring *E. coli* cell concentration, ${\text{O}}_{2}$ concentration, and fluid flows, we found that this self-organization emerges from a feedback mechanism where cellular motility both responds to and shapes ${\text{O}}_{2}$ gradients (Fig. 3*A*). By integrating our experiments with theoretical modeling and numerical simulations, we have established a quantitative biophysical framework that describes how this emergent behavior depends on system parameters, such as the droplet size and cell concentration (Fig. 3*I*). Our study therefore complements and extends prior work that either mapped ${\text{O}}_{2}$ gradients in nonmotile bacterial colonies (29), or explored their consequences in motile suspensions under the influence of gravity, which gives rise to distinct fascinating phenomena (63, 86, 87). Our work also helps to frame questions and research directions that build on our approach. For example, owing to the generality of our theoretical framework, it could be extended to other organisms and even mixed systems with multiple cell types and limiting resources (88–93).

### Implications for Natural Bacterial Populations.

Aerotaxis is typically thought of as a mechanism by which bacteria can find the most favorable ${\text{O}}_{2}$ conditions for survival and growth (57). Our work suggests another potential biological function imparted by aerotaxis: it enables cells to reshape their entire population by modulating ${\text{O}}_{2}$ fluxes. For example, we found that under conditions of “transient anoxia” (gray-orange in Fig. 3*I*), bacteria use aerotaxis to accumulate at the air–liquid interface and augment ${\text{O}}_{2}$ influx (Fig. 2*C*)—enabling cells from the core to eventually recover motility. Therefore, while aerotaxis likely evolved to benefit individual bacteria, exploring the biological implications of this phenomenon, such as for population fitness, will be an interesting direction for future research. Indeed, it could be a previously unrecognized strategy for bacterial populations to optimize their collective resource utilization under confinement. It will also be interesting to explore the biological implications of the core–shell organization revealed by our work more broadly. For example, the formation of an anoxic core could influence other bacterial processes beyond motility—such as quorum sensing (37, 63) and biofilm formation (39)—over long time scales.

Our results could be particularly relevant for understanding bacterial behavior in natural settings, ranging from soils and sediments (94–96) to gels and tissues in the body (5, 17, 37, 72, 97, 98), where cells often experience both spatial confinement and resource limitation (99). In human health, our results may help shed light on the organization of bacteria in ${\text{O}}_{2}$-limited niches within the body, such as in mucus or chronic wounds, where populations are often layered, as in our experiments. It could be that such hierarchical structures protect a subpopulation of the cells from predators like phages and neutrophils (100) and external stressors such as antibiotics (101, 102); however, other factors such as access to other metabolites, e.g., carbon, mechanical interactions, and other immune responses will also play important roles. In environmental science, our findings could help explain bacterial distribution patterns in soil micropores, with potential implications for understanding biogeochemical cycles. The spatial organization we observe could also influence key processes such as carbon mineralization in soil aggregates or nitrogen cycling in marine snow particles, where bacterial activity is often limited by ${\text{O}}_{2}$ availability. Additionally, the principles uncovered by our work could help guide ways to control bacterial populations for practical applications in biotechnology. For example, our findings could be leveraged to design droplet-based bacterial bioreactors or cultivation systems whose spatial organization is controlled to optimize desired metabolic processes (e.g., biofuel synthesis, protein production) while maintaining high cell concentrations and avoiding ${\text{O}}_{2}$ limitations.

### Implications for Active Matter Physics.

Our work establishes principles governing pattern formation in active matter systems—in particular, collectives of actively moving particles, exemplified in our case by motile bacteria, with spatially varying activity. These are often studied within the context of motility-induced phase separation (MIPS), in which the slowing of particles when they are more concentrated causes them to accumulate, forcing them to spontaneously separate into dilute and concentrated phases (103, 104). Our finding that in a large, concentrated droplet, its core acts as a “motility sink” for cells is reminiscent of this phenomenon. However, in typical models of MIPS, the concentration dependence of particle mobility arises directly from purely mechanical interactions between particles, whereas in our case, it arises indirectly via a feedback loop where the spatial distribution of mobility both responds to and shapes the chemical field that controls it. Incorporating such chemically mediated feedback could be an interesting extension of MIPS, as well as other continuum models of active matter.

This study also provides a platform to examine the dynamics of active–passive interfaces (e.g., by monitoring the motile shell-immotile core interface), which has previously been studied only in the context of purely mechanical interactions (105–109). The additional chemical coupling in our system could introduce additional effects at these interfaces arising from the interplay between aerotaxis (46), shear-induced depletion (110, 111), and cell–cell hydrodynamic interactions. Such effects might culminate in the emergence of directed long-range flows that can transport nutrients or signaling molecules (112, 113) more efficiently than diffusion (64, 114, 115).

## Materials and Methods

For each experiment, we grow an overnight culture of fluorescent *E. coli* (W3110) in LB medium at 30 ^°^C under shaking conditions. A 1% inoculum is then incubated in fresh LB for 3 h until the optical density (measured using a Biowave Cell Density Meter CO8000) reaches ∼0.5. The cells are washed three times with motility buffer (BMB) and prepared at the desired concentration for a given experiment. This process involves centrifuging the cells at 700 to 1,200×*g* (where g represents standard gravitational acceleration), which is sufficiently low to minimally affect flagella as shown previously (116, 117). Indeed, our observation that regions of the annular shell are in some cases completely devoid of cells (e.g., Fig. 4) that have moved either into the anoxic core or to the air–liquid interface confirms that the cells all retain the ability to swim. Finally, as a negative control for motility, we perform the same experiment as in Fig. 1F but with a nonmotile mutant strain that lacks flagella (*E. coli* W3110 ΔflhDC). As shown in *SI Appendix*, unlike the motile case, the nonmotile suspension does not undergo core/shell spatial organization, but remains uniformly dispersed, indicating that motility is essential.

The test cell consists of two parallel glass coverslips, cleaned with ethanol before use, separated by a 130μm-thick air-permeable Parafilm spacer. A droplet of bacterial suspension is deposited on the bottom coverslip, with the volume controlled to reach a desired droplet diameter, with microdroplets of deionized water added around it to maintain humidity and prevent evaporation. The system is sealed with a second overlying coverslip, allowing oxygen to diffuse only through the Parafilm, which is commonly used in microbiology as an oxygen-permeable film. There is an additional air gap surrounding the droplet and the inner edge of the spacer ≈9 to 15 mm wide, which provides additional oxygen influx. This ∼50 μL airspace contains enough oxygen for all the cells over the experimental duration, even without the additional influx through the surrounding Parafilm spacer.

There is inevitably some slight evaporation in the experiments. Measuring the resulting reduction in the droplet diameter enables us to estimate a characteristic convective flow speed $v_{c}\approx 0.05$ μm/s, which is 500× slower than the bacterial swimming speed. The associated Péclet number for O_2_ transport is $\mathrm{Pe}=\frac{v_{c}L}{D_{O_{2}}}∼10^{-2}\;\mathrm{to}\;10^{-1}\ll 1$, with the characteristic length L ranging from 130μm (the gap height beyond which any hydrodynamic flows initiated at the interface are screened by confinement) to 3.5mm (the largest droplet radius, used as an upper bound). Therefore, any slight convection due to droplet evaporation is far too weak to influence bacterial swimming or O_2_ transport.

We start imaging approximately one minute after the droplet flattens into a disk-like shape, using a Nikon A1R+ inverted laser scanning confocal microscope with a temperature-controlled stage maintained at 30 ^°^C. Population dynamics are imaged at 1 frame per second using a 4× objective, capturing both bright-field and fluorescence images from an optical slice of ∼100 μm thickness. For the state diagram in Fig. 3*I*, multiple droplets are imaged using a 4× objective, with large-area scanning via stitched frames obtained at 0.04 frames per second. Flow patterns are visualized using a 20× objective at 30 frames per second from a ∼10 μm optical slice. Dissolved oxygen concentration is measured using a ruthenium complex (Ru(dpp)) whose fluorescence (emission ∼ 615 nm) is quenched upon oxygen binding. To mitigate toxicity to *E. coli*, the dye is encapsulated in DMPC-PEG2000 phospholipid micelles. This dye was independently validated for conditions similar to those tested in our experiments previously (44, 61), and we directly follow the protocol of this previous work in preparation and calibration of the dye. We quantify oxygen concentration via the measured fluorescence intensity, calibrated according to the calibration curve reported by Douarche et al. (61), described by the equation $I_{\text{fluo}}=1+6.5\cdot e^{-p{\text{O}}_{2}/3}$, where $I_{\text{fluo}}$ is the fluorescence intensity normalized by the fluorescence signal at the saturated concentration and $p{\text{O}}_{2}$ is the oxygen partial pressure. The dissolved oxygen concentration $c_{{\text{O}}_{2}}$ is obtained from the partial pressure values via Henry’s law. As shown in *SI Appendix*, we verify that photobleaching or biochemical factors associated with microbial activity do not change the fluorescence intensity associated with the O_2_ probe.

We use the Beer–Lambert law to calibrate cell concentration in the bright-field images, use particle image velocimetry (PIV) in PIVLab to measure the velocity field generated by the swimming cells, and track individual cells using the ImageJ plugin TrackMate. Due to the noncircular shape of some droplets, we calculate the average intensity profile within a narrow sector of the droplet to obtain the kymographs of cell concentration.

## Supplementary Material

Appendix 01 (PDF)

Movie S1. Population dynamics for a small droplet containing a dilute suspension of bacteria. (Corresponds to main text Fig. 1b.)

Movie S2. Population dynamics for a large droplet containing a concentrated suspension of bacteria. (Corresponds to main text Fig. 1d.)

Movie S3. High magnification movie of the bacterial motility dynamics within the core and the annulus for a small droplet containing a concentrated bacterial suspension. (Corresponds to main text Fig. 1d magnified panel.)

Movie S4. Population dynamics for a small droplet containing a concentrated suspension of bacteria. (Corresponds to main text Fig. 1f.)

Movie S5. Simultaneous visualization of the bacterial dynamics (darker means higher cell concentration) and the oxygen concentration field (darker means higher oxygen concentration). The movie is associated with a small concentrated droplet. (Corresponds to main text Fig. 2d.)

Movie S6. Bacterial accumulation at the air-liquid interface due to aerotaxis. (Corresponds to main text Fig. 2c.)

Movie S7. Population dynamics for a small droplet containing a dilute suspension of bacteria obtained by simulations. (Corresponds to main text Fig. 3b.)

Movie S8. Population dynamics for a large droplet containing a concentrated suspension of bacteria obtained by simulations. (Corresponds to main text Fig. 3d.)

Movie S9. Population dynamics for a small droplet containing a concentrated suspension of bacteria obtained by simulations. (Corresponds to main text Fig. 3f.)

Movie S10. Population dynamics for a small droplet containing a concentrated suspension of bacteria obtained by simulations. Here, we have increased the diffusion coefficient and the aerotactic sensitivity of bacteria by a factor of 4 which is comparable to values in the viscoelastic fluid case.

Movie S11. Population dynamics for a small viscoelastic droplet containing a concentrated suspension of bacteria. The continuous phase is an aqueous polymeric solution PEO 5 MDa *c*_pol_ = 0.2wt.%. (Corresponds to main text Fig. 4.)

Movie S12. Left: Dimensionless bacterial and O_2_ concentrations as a function of the dimensionless radius over time, obtained by simulations. Right: Dimensionless O_2_ concentration, O_2_ influx, and cell concentration at the droplet interface as functions of dimensionless time. Here, ${\tilde{k}}_{t}=0.1$ and $\tilde{R}=3.9$.

Movie S13. Same as Movie S12 but for ${\tilde{k}}_{t}=10$ and $\tilde{R}=27$.

## Data Availability

All movies and analysis codes are available at Zenodo (https://doi.org/10.5281/zenodo.14894704) (118). The code used to solve the coupled reaction–diffusion equations describing bacteria and oxygen dynamics in the manuscript are available at https://github.com/amcalv/2025-Code-Spatial-self-organization-of-confined-bacterial-suspensions (119). All other data are included in the manuscript and/or supporting information.
