# Supplementary material for: Spatial self-organization of confined bacterial suspensions
Source: Proc Natl Acad Sci U S A. 2025 Oct 6;122(41):e2503983122. doi: 10.1073/pnas.2503983122 (PMC12541401; doi:10.1073/pnas.2503983122)
Supplement: Supplementary file 1 — Appendix 01 (PDF) [file pnas.2503983122.sapp.pdf]

# Supplementary Information: Spatial self-organization of confined bacterial suspensions

**Babak Vajdi Hokmabad**[a], **Alejandro Martínez-Calvo**[a,b], **Sebastian Gonzalez La Corte**[c], **and Sujit S. Datta**[d,a,1]

[a]Department of Chemical and Biological Engineering, Princeton University, Princeton, NJ 08544; [b]Princeton Center for Theoretical Science, Princeton University, Princeton, NJ 08544; [c]Lewis-Sigler Institute for Integrative Genomics, Princeton University, Princeton, NJ 08540; [d]Division of Chemistry and Chemical Engineering, California Institute of Technology, Pasadena, CA 91125

**Non-dimensionalization of the model.** To identify the key governing parameters of our model, we make Eqs. (1a)-(1c) in Fig. 3a dimensionless. To this end, we choose the $O_2$ penetration length $\ell_{O_2} = \sqrt{D_{O_2} c_{O_2,\mathrm{sat}}/(k_{O_2,0} c_{\mathrm{cell},0})}$, which arises from a balance between $O_2$ diffusion and cell $O_2$ uptake, as the characteristic length scale. As characteristic $O_2$ concentration and cell density we choose the $O_2$ saturation concentration, $c_{O_2,\mathrm{sat}}$, and the initial cell number concentration, $c_{\mathrm{cell},0}$, respectively. As characteristic time scale, we consider the time it takes for cells to migrate via aerotaxis over the $O_2$ penetration length scale, i.e., $t_c = \ell_{O_2}^2/\chi_0$. Introducing these characteristic scales, the dimensionless conservation equations for bacteria and $O_2$ read:

$$\text{Bacteria}: \quad \partial_{\tilde{t}} \tilde{c}_{\mathrm{cell}} = \boldsymbol{\nabla} \cdot [\tilde{D}_{\mathrm{cell}}(\tilde{c}_{O_2}) \boldsymbol{\nabla} \tilde{c}_{\mathrm{cell}} - \tilde{\chi}(c_{O_2}) \tilde{c}_{\mathrm{cell}} \boldsymbol{\nabla} \tilde{f}(\tilde{c}_{O_2})], \tag{S1a}$$

$$\text{Oxygen}: \quad \Gamma \partial_{\tilde{t}} \tilde{c}_{O_2} = \boldsymbol{\nabla}^2 \tilde{c}_{O_2} - \tilde{k}_{O_2}(\tilde{c}_{O_2}) \tilde{c}_{\mathrm{cell}} \frac{\tilde{c}_{O_2}}{\tilde{c}_{O_2} + \tilde{K}}, \tag{S1b}$$

where $\tilde{D}_{\mathrm{cell}}(\tilde{c}_{O_2}) = \frac{\tilde{D}_{\mathrm{cell},0}}{2}(1 + \tanh[(\tilde{c}_{O_2} - \tilde{c}_{\mathrm{crit}})/\tilde{\delta}])$, $\tilde{\chi}(\tilde{c}_{O_2}) = \frac{1}{2}(1 + \tanh[(\tilde{c}_{O_2} - \tilde{c}_{\mathrm{crit}})/\tilde{\delta}])$, $\tilde{k}_{O_2}(\tilde{c}_{O_2}) = \frac{1}{2}(1 + \tanh[(\tilde{c}_{O_2} - \tilde{c}_{\mathrm{crit}})/\tilde{\delta}])$, and $\tilde{f}(\tilde{c}_{O_2}) = \tilde{c}_{O_2}/(\tilde{K}_\chi + \tilde{c}_{O_2})$. We impose radial symmetry at $\tilde{r} = 0$, no flux of bacteria across the liquid-air interface at $\tilde{r} = \tilde{R}$, and the dimensionless form of the $O_2$ interfacial transport equation (Eq. 1b in Fig. 3a), given by:

$$\boldsymbol{\nabla} \tilde{c}_{O_2} \cdot \hat{\boldsymbol{e}}_{\tilde{r}} = \tilde{k}_t(\tilde{c}_{O_2} - 1) \quad \text{at } \tilde{r} = \tilde{R}. \tag{S2}$$

The dimensionless parameters that emerge in Eq. (S1) and Eq. (S2) are:

$$\tilde{R} \equiv \frac{R}{\ell_{O_2}} = \frac{\text{Droplet radius}}{O_2 \text{ penetration length}}, \tag{S3}$$

$$\tilde{k}_t \equiv \frac{k_t \ell_{O_2}}{D_{O_2}} = \frac{O_2 \text{ interfacial transport rate}}{O_2 \text{ diffusive rate}}, \tag{S4}$$

$$\Gamma \equiv \frac{\chi_0}{D_{O_2}} = \frac{\text{Bacterial aerotaxis}}{O_2 \text{ diffusion}}, \tag{S5}$$

$$\tilde{D}_{\mathrm{cell},0} \equiv \frac{D_{\mathrm{cell},0}}{\chi_0} = \frac{\text{Bacterial diffusion}}{\text{Bacterial aerotaxis}}, \tag{S6}$$

$$(\tilde{K}, \tilde{K}_\chi, \tilde{c}_{\mathrm{crit}}, \tilde{\delta}) \equiv \frac{(K, K_\chi, c_{\mathrm{crit}}, \delta)}{c_{O_2,\mathrm{sat}}} = \frac{O_2 \text{ characteristic concentrations}}{O_2 \text{ saturation concentration}}. \tag{S7}$$

We emphasize that our numerical simulations, shown in Fig. 3 of the main text, use experimental measurements for all parameters in the model; direct measurements of the parameters relating to bacterial aerotaxis and $O_2$ transport were not possible in our experimental setup, so we used the values from other

experimental measurements reported previously. The values of all dimensional and dimensionless parameters used in the numerical simulations are reported in Table S2. The parameter $\tilde{\delta}$, which measures the sharpness of the motility loss transition around the dimensionless critical $O_2$ concentration, $\tilde{c}_{\mathrm{crit}}$, is always set to $\tilde{\delta} = 8 \times 10^{-7}$.

We do not consider any enhancement in $O_2$ diffusion by cellular motility, even though it is well established that swimming bacteria can enhance the diffusivity of a passive solute by stirring the surrounding fluid (1–3). However, as established previously (1–4), the magnitude of this enhancement is highly sensitive to the size of the solute, the bacterial swimming speed, and the length scale over which coherent flows are generated by bacterial swimming. Indeed, these prior studies focused on settings in which the solute is $\sim 10^1 - 10^4\times$ larger than molecular $O_2$, bacterial swimming is $\gtrsim 5\times$ faster, or the coherence length of the flows generated by the cells is $\gtrsim 2\times$ larger than in our experiments—significantly enhancing this effect compared to our study. As shown previously (4), as the solute becomes smaller than $\sim 400$ nm ($\sim 10^4\times$ larger than $O_2$), diffusive dynamics increasingly dominate over advective effects induced by cellular swimming; therefore, we expect that cell swimming only minimally enhances $O_2$ diffusion in our experiments. This expectation is corroborated by calculating the Péclet number associated with $O_2$ transport across the length $L \approx 30$ μm that characterizes the size of the coherent vortices observed in our concentrated *E. coli* suspensions: $\mathrm{Pe} = \frac{U\ L}{D_{O_2}} \approx 0.2 < 1$, indicating that even at the highest bacterial concentrations—where swimming-generated flow is the strongest—oxygen transport in our system is minimally affected by cellular swimming. This expectation is also corroborated by our experimental measurements in Fig. 2B of the main text, which agree well with our theoretical calculation without needing to modify the $O_2$ diffusion coefficient. However, as an additional test of this point, we repeat the simulation described in the main text, but with a cell density-dependent diffusivity given by $D_{O_2}(c_{\mathrm{cell}}) = D_{O_2,\mathrm{min}} + D_{O_2,\mathrm{min}} c_{\mathrm{cell}}/(c_{\mathrm{cell}} + c_{1/2})$ for $c_{O_2} > c_{\mathrm{crit}}$, where $D_{O_2}$ increases linearly with $c_{\mathrm{cell}}$ at low cell concentration and saturates to a maximum value of $2D_{O_2,\mathrm{min}}$ at high enough cell concentration (motivated by prior work reporting a $\sim 2 - 4\times$ enhancement), $c_{1/2}$ is a characteristic cell concentration, and $c_{\mathrm{crit}}$ is the critical $O_2$ concentration below which cells lose motility. As shown in Fig. S7, which corresponds to Figs. 3D and F of the main text, the essential features of the simulations are still preserved in both cases. We observe a slight enhancement in cellular accumulation at the air-liquid interface, and a slightly smaller immotile core, but the same physical picture as in the main manuscript still holds. Taken altogether, these results indicate that while $O_2$ diffusion may indeed be slightly enhanced by cellular swimming, this effect is minimal for the conditions explored in our experiments, and our minimal model that does not incorporate such enhancement is sufficient to capture the essential features of the experiments.

Hydrodynamic interactions generated by the swimming cells exist in both Newtonian- and non-Newtonian fluids if the suspension is concentrated enough. However, we chose to neglect them from the model for two key reasons. First, there is a large separation of time scales in our system: The central focus of our study is on the spatial core/shell self-organization of the bacterial suspension over time scales $\gtrsim 1$ h, while hydrodynamic interactions manifest at time scales of seconds or less, $\gtrsim 10^3\times$ shorter. Therefore, our model focuses on the slower processes — oxygen transport and availability, cellular respiration, and motility-induced changes in cellular distribution — that dominate the long-term behavior that we focus on here. Second, given that our focus is on the spatial self-organization of the bacterial suspension, we sought to develop the most parsimonious model that can explain this phenomenon. While including hydrodynamic interactions would likely improve the quantitative agreement between the model predictions and the experimental measurements, it would considerably increase the complexity of the model. Indeed, in concentrated bacterial suspensions, hydrodynamic interactions involve multi-body effects that are difficult to model accurately without substantial computational resources. By contrast, our minimal model provides a tractable numerical implementation, and yields analytical solutions that provide mechanistic insights.

[1]To whom correspondence should be addressed. E-mail: ssdatta@caltech.edu

Our model does not consider any variations in $z$. Indeed, we chose the experimental droplet height to be 130 µm to minimize any variations in $z$. Prior work (5) established that the cell concentration $c_{\text{cell}}$ varies exponentially in $z$ due to competition between their sedimentation and active swimming: $c_{\text{cell}}(z)/c_{\text{cell}}(z=0) = e^{-z/\ell_g}$ where the gravitational height $\ell_g = D_{\text{cell}}/v_{\text{s}}$, $D_{\text{cell}} \approx 200$ µm$^2$s$^{-1}$ is the active cellular diffusivity, and $v_{\text{s}} \approx 0.1$ µm s$^{-1}$ is the cell sedimentation speed, yielding $\ell_g \sim 2$ mm $\geq 15\times$ larger than the height of our droplets. Therefore, we expect $z$ variations to be minimal. Prior high-resolution optical coherence tomography (OCT) measurements (3) confirmed this expectation. Moreover, our own direct measurements of the cell swimming-generated flow at different depths ($h = 20, 30,$ and 40 µm from the bottom surface shown in panels A, B, and C of Fig. S8, respectively) all show similar results, supporting this point.

We find excellent quantitative agreement between the model and the data in the non-dimensionalized phase diagram of Fig. 3I of the main text — which summarizes the results of $> 250$ different experiments. In addition, we find reasonable quantitative agreement between the representative model predictions shown in main text Figs. 3B-G & H and the representative experimental measurements in Fig. 1B-G & 2B; the slight discrepancy (still with agreement within a factor of $\approx 2$) between the predicted and measured values of the length scale $\ell_{\text{init}}$ likely reflects the influence of other complexities, such as cell-cell interactions and hydrodynamic effects, that we neglect from our model for simplicity. The goal of our minimal model is not to incorporate all the complexities at play in the experiments, but to isolate the key mechanisms driving the spatial self-organization of the bacterial population that our manuscript focuses on. Incorporating these additional effects may improve quantitative agreement further, and will be a useful future extension of our work.

For the same bacterial species and liquid medium, the only dimensionless parameters that vary in experiments are $\tilde{R}$ and $\tilde{k}_{\text{t}}$. In particular, the parameter $\tilde{k}_{\text{t}}$, which only enters in the model through the liquid-air boundary condition Eq. (S2), exhibits interesting limiting cases. (i) When $\tilde{k}_{\text{t}} \gg 1$, the dimensionless $O_2$ concentration at the interface remains constant, i.e., $\tilde{c}_{O_2} = 1$, which implies $c_{O_2} = c_{O_2,\text{sat}}$. In this limiting case, the interfacial influx of $O_2$ is faster than $O_2$ diffusion and bacterial $O_2$ uptake, so that $c_{O_2}$ always equilibrates to its saturation value (see Movie S12). However, the influx of $O_2$ across the interface is not zero—it adjusts over time to maintain the saturation level of $O_2$ at all times. This corresponds to the limit approaching the left in the state diagram in Fig. 3. (ii) When $\tilde{k}_{\text{t}} \ll 1$, $\boldsymbol{\nabla}\tilde{c}_{O_2} \cdot \hat{\boldsymbol{e}}_{\tilde{r}} = 0$ at $\tilde{r} = \tilde{R}$, meaning that the influx of $O_2$ at the interface is significantly slower than bacterial $O_2$ uptake, effectively imposing a no-flux boundary condition (see Movie S13). This limit corresponds to, for example, a scenario with a high density of bacteria that consume $O_2$ very efficiently, such that interfacial $O_2$ transport cannot keep up. In this case, permanent anoxia should always develop as $O_2$ eventually depletes. This corresponds to the limit approaching the right in the state diagram in Fig. 3.

**Supplementary movies.** All movies are available at 10.5281/zenodo.14894704.

**Movie S1.** Population dynamics for a small droplet containing a dilute suspension of bacteria. (Corresponds to main text Fig. 1b.)

**Movie S2.** Population dynamics for a large droplet containing a concentrated suspension of bacteria. (Corresponds to main text Fig. 1d.)

**Movie S3.** High magnification movie of the bacterial motility dynamics within the core and the annulus for a small droplet containing a concentrated bacterial suspension. (Corresponds to main text Fig. 1d magnified panel.)

**Movie S4.** Population dynamics for a small droplet containing a concentrated suspension of bacteria. (Corresponds to main text Fig. 1f.)

**Movie S5.** Simultaneous visualization of the bacterial dynamics (darker means higher cell concentration) and the oxygen concentration field (darker means higher oxygen concentration). The movie is associated with a small concentrated droplet. (Corresponds to main text Fig. 2d.)

**Movie S6.** Bacterial accumulation at the air-liquid interface due to aerotaxis. (Corresponds to main text Fig. 2c.)

**Movie S7.** Population dynamics for a small droplet containing a dilute suspension of bacteria obtained by simulations. (Corresponds to main text Fig. 3b.)

**Movie S8.** Population dynamics for a large droplet containing a concentrated suspension of bacteria obtained by simulations. (Corresponds to main text Fig. 3d.)

**Movie S9.** Population dynamics for a small droplet containing a concentrated suspension of bacteria obtained by simulations. (Corresponds to main text Fig. 3f.)

**Movie S10.** Population dynamics for a small droplet containing a concentrated suspension of bacteria obtained by simulations. Here, we have increased the diffusion coefficient and the aerotactic sensitivity of bacteria by a factor of 4 which is comparable to values in the viscoelastic fluid case.

**Movie S11.** Population dynamics for a small viscoelastic droplet containing a concentrated suspension of bacteria. The continuous phase is an aqueous polymeric solution PEO 5 MDa $c_{\mathrm{pol}} = 0.2$wt.%. (Corresponds to main text Fig. 4.)

**Movie S12.** Left: Dimensionless bacterial and $O_2$ concentrations as a function of the dimensionless radius over time, obtained by simulations. Right: Dimensionless $O_2$ concentration, $O_2$ influx, and cell concentration at the droplet interface as functions of dimensionless time. Here, $\tilde{k}_{\mathrm{t}} = 0.1$ and $\tilde{R} = 3.9$.

**Movie S13.** Same as Movie S12 but for $\tilde{k}_{\mathrm{t}} = 10$ and $\tilde{R} = 27$.


1. MJ Kim, KS Breuer, Enhanced diffusion due to motile bacteria. *Phys. fluids* **16**, L78–L81 (2004).
2. A Jepson, VA Martinez, J Schwarz-Linek, A Morozov, WC Poon, Enhanced diffusion of nonswimmers in a three-dimensional bath of motile bacteria. *Phys. Rev. E* **88**, 041002 (2013).
3. A Sokolov, RE Goldstein, FI Feldchtein, IS Aranson, Enhanced mixing and spatial instability in concentrated bacterial suspensions. *Phys. Rev. E* **80**, 031903 (2009).
4. AJ Mathijssen, R Jeanneret, M Polin, Universal entrainment mechanism controls contact times with motile cells. *Phys. Rev. Fluids* **3**, 033103 (2018).
5. J Schwarz-Linek, et al., Escherichia coli as a model active colloid: A practical introduction. *Colloids Surfaces B: Biointerfaces* **137**, 2–16 (2016).
6. C Douarche, A Buguin, H Salman, A Libchaber, E. Coli and Oxygen: A Motility Transition. *Phys. Rev. Lett.* **102**, 198101 (2009).
7. HC Berg, *Random Walks in Biology.* (1993).
8. M Rubinstein, RH Colby, *Polymer Physics.* (Oxford University Press), (2003).
9. WW Graessley, Polymer chain dimensions and the dependence of viscoelastic properties on concentration, molecular weight and solvent power. *Polymer* **21**, 258–262 (1980).
10. AI Flamholz, S Saccomano, K Cash, DK Newman, Optical o2 sensors also respond to redox active molecules commonly secreted by bacteria. *Mbio* **13**, e02076–22 (2022).
11. J Bouvard, C Douarche, P Mergaert, H Auradou, F Moisy, Direct measurement of the aerotactic response in a bacterial suspension. *Phys. Rev. E* **106**, 034404 (2022).
12. X Fu, et al., Spatial self-organization resolves conflicts between individuality and collective migration. *Nat. Commun.* **9**, 2177 (2018).
13. A Martínez-Calvo, et al., Morphological instability and roughening of growing 3D bacterial colonies. *Proc. Natl. Acad. Sci.* **119**, e2208019119 (2022).
14. J Shioi, CV Dang, BL Taylor, Oxygen as attractant and repellent in bacterial chemotaxis. *J. Bacteriol.* **169**, 3118–3123 (1987).
15. JR Hazel, BD Sidell, A method for the determination of diffusion coefficients for small molecules in aqueous solution. *Anal. Biochem.* **166**, 335–341 (1987).
16. RG Wetzel, *Limnology: lake and river ecosystems.* (gulf professional publishing), (2001).
17. SG Schladow, M Lee, BE Hürzeler, PB Kelly, Oxygen transfer across the air-water interface by natural convection in lakes. *Limnol. Oceanogr.* **47**, 1394–1404 (2002).

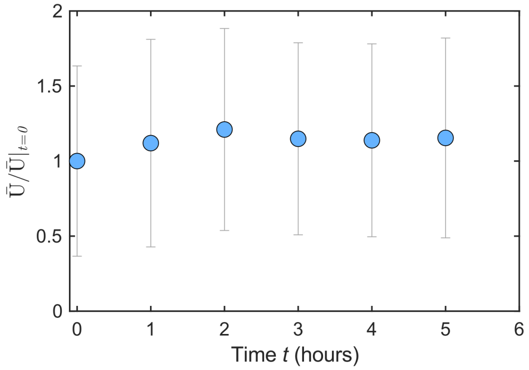


**Fig. S1. Mean speed of bacteria measured in BMB over 5 hours.** We track individual bacteria in a dilute suspension with $c_{\mathrm{cell},0} = 10^8\ \mathrm{cells/mL}$ every hour, over a period of five hours. As shown by the data, the mean swimming speed BMB does not change, indicating that the cells have enough endogenous energy resources to continue swimming over this duration, consistent with prior measurements (5). The error bars correspond to the standard deviation of the measured speed distribution.

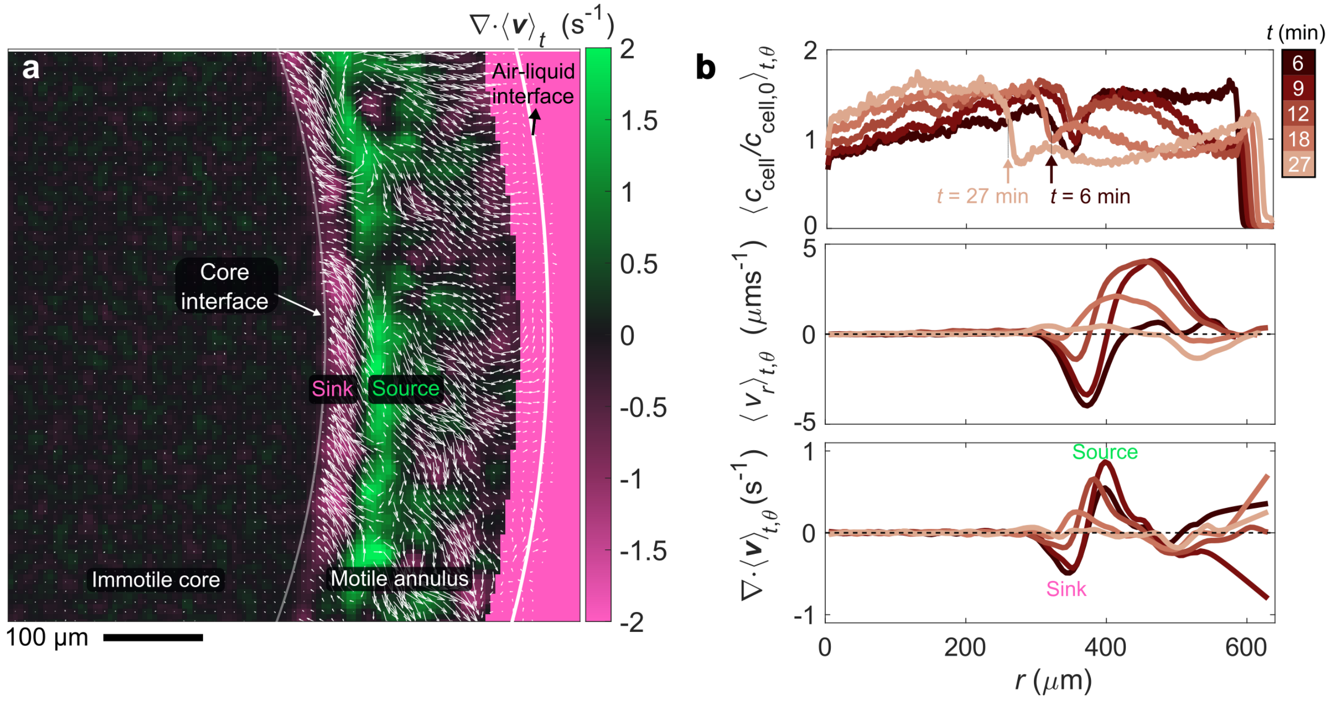


**Fig. S2. Mean flow field generated by swimming bacteria in the annulus. a,** Mean flow field generated for $c_{\mathrm{cell},0} = 8 \times 10^{10} \mathrm{cells/mL}$ measured at a height of $30\ \mu\mathrm{m}$ from the bottom surface. The background colormap shows the divergence of the mean velocity field $\nabla \cdot \langle v \rangle_{t,\theta}$. **b** Time evolution of the normalized cell concentration $\langle c_{\mathrm{cell}}/c_{\mathrm{cell},0} \rangle_{t,\theta}$ (top), the mean radial component of the velocity $\langle v_r \rangle_{t,\theta}$ (middle), and the divergence of the mean velocity field $\nabla \cdot \langle v \rangle_{t,\theta}$ (bottom). The mean velocity field and all the mean profiles are obtained by averaging over 1 minute (1800 frames). The arrows in the top panel show the location of the core boundary at $t = 6$ and $27$ minutes after the start of the experiment. The divergence of the velocity field shows a positive value at a radial distance $100\ \mu\mathrm{m}$ away from the core boundary. This observation indicates that there is a vertical top-down flow, acting as a *source* of cells, that splits into two radial streams close to the bottom surface. The stream between the *source* and the core is directed towards the core ($\langle v_r \rangle_t < 0$) where they lose their motility after $\tau_{\mathrm{delay}} \approx 5$ minutes (6), sediment and locally increase the $c_{\mathrm{cell}}$. In this part of the annulus, we observe $\boldsymbol{\nabla} \cdot \langle \boldsymbol{v} \rangle_t < 0$, *i.e.,* the core is acting as another *sink* for cells. The opposite stream moves in the radial direction towards the air-liquid interface ($\langle v_r \rangle_t > 0$) resulting in an accumulation at the interface again acting as a *sink* for the flux of bacteria. Thus, with sinks pulling cells both towards the core and the air-liquid interface, the middle part of the annulus gradually becomes depleted of bacteria.

| $c$ (w/w%) | $\bar{v}$ ($\mu$m/sec) | $\bar{\tau}$ (sec) | $D$ ($\mu$m$^2$/sec) |
|---|---|---|---|
| 0 | 25 | 0.6 | 187 |
| 0.1 | 40 | 0.6 | 480 |
| 0.15 | 38 | 0.6 | 433 |
| 0.2 | 43 | 0.8 | 725 |

**Table S1. Diffusion coefficient of individual cells at different PEO concentrations.** We first prepare an overnight culture of bacteria. Next, these bacteria are re-suspended at a more dilute concentration of 3 to 5 $\times 10^5$ cells/mL in the polymer solution of choice. Next, the suspension of cells is put between two glass slides separated by paraffin film. The cells are then imaged with the confocal microscope with a $10\times$ air objective at a temperature of $30°$C. We acquire 512 by 512 pixel images at $\approx$ 33 ms time intervals. We use the ImageJ plugin TrackMate to track bacteria and we write a custom MATLAB script to extract speed and running time distributions. From these distributions we compute the average bacterial speed, $\bar{v}$, and average run duration $\bar{\tau}$. We then estimate the diffusion coefficient as $D \approx \bar{v}^2 \bar{\tau}/2$ following (7).

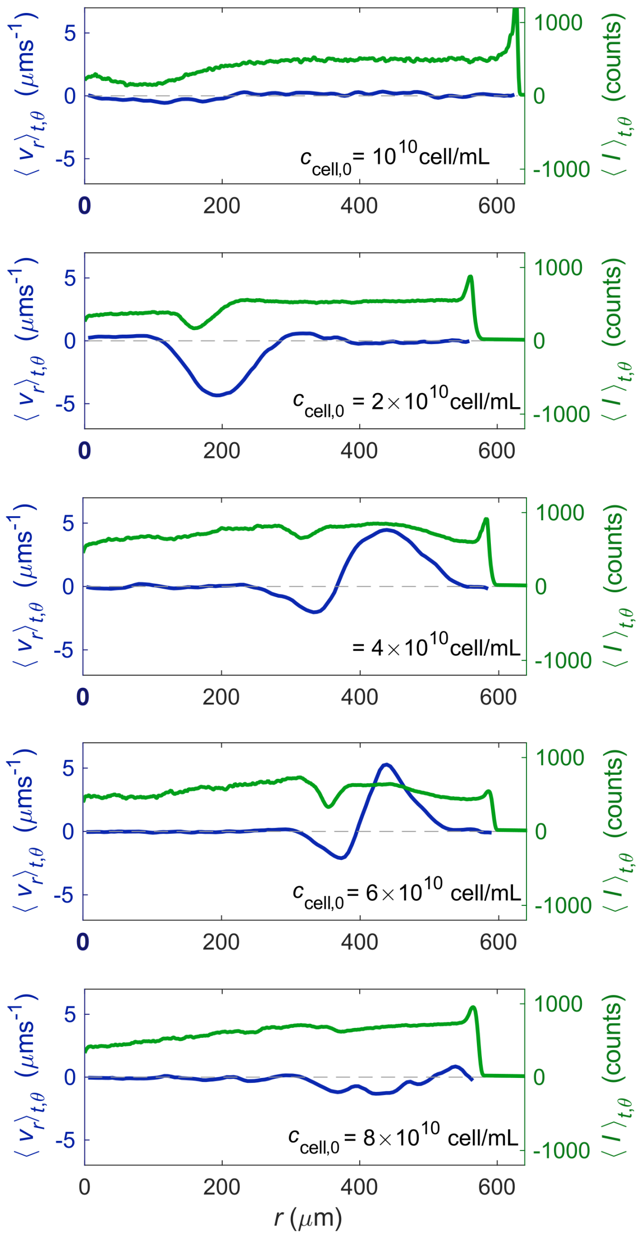


**Fig. S3. Effect of cell concentration on the mean flow field generated by bacteria.** The left axis shows the radial component of the velocity field plotted against the radial direction. On the right axis, the instantaneous GFP signal intensity profiles are displayed in light grey, with the mean intensity profile highlighted in orange. Since intensity is proportional to cell concentration, the small dip in the intensity profile indicates the edge of the core. The mean profiles are calculated by averaging data recorded during the first two minutes following core formation.

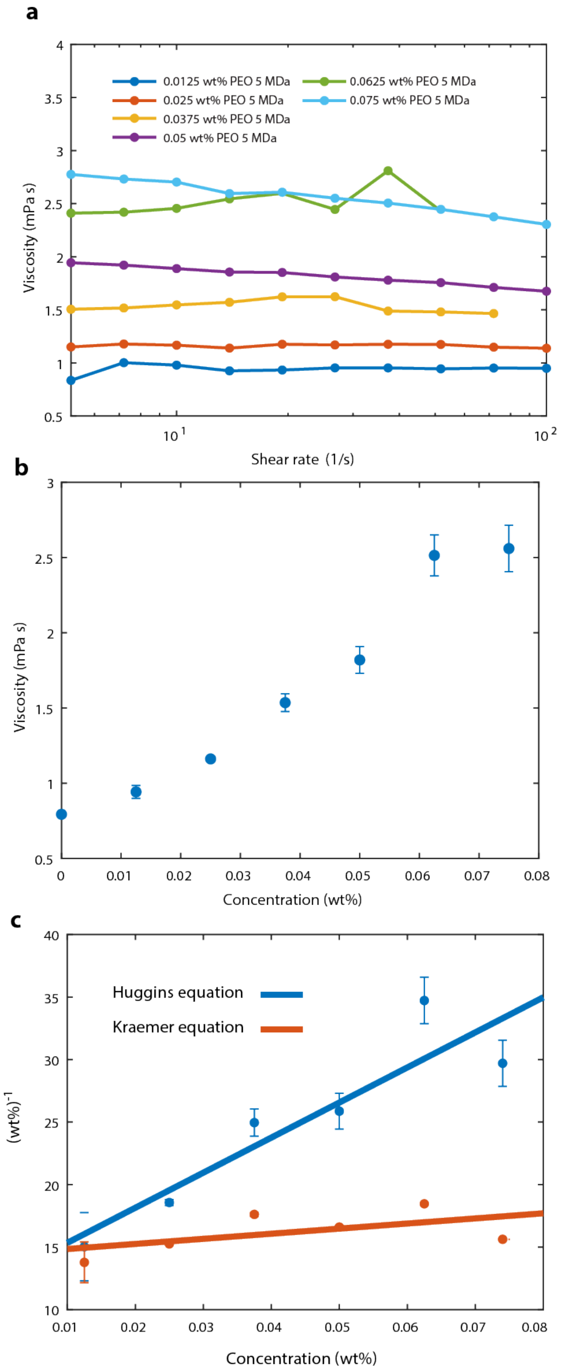


**Fig. S4. Rheological characterization of polymer solutions.** We use an Anton Paar MCR501 rheometer with a double-gap Couette geometry to characterize our polymer solutions. **a** We measure the dynamic shear viscosity as a function of imposed shear rate; as shown by the data, for the polymer concentrations used in this study we observe shear-thinning behavior. **b** Low shear rate viscosity vs. concentration curve. The dots represent the mean viscosity measured over all shear rates at constant polymer concentration, while the error bars correspond to the variation of the viscosity at constant polymer concentration over the range of shear rates used, $5 - 200$ 1/s. This dependence of viscosity on polymer concentration $c$ yields a straightforward way to determine the polymer overlap concentration $c^*$. In particular, writing the viscosity as a virial expansion $\eta = \eta_{\mathrm{s}} \left(1 + [\eta]\, c + k_{\mathrm{H}}\, [\eta]^2\, c^2 + \ldots\right)$ and retaining only the first three terms on the right hand side yields two distinct relations (8); here, $[\eta]$ is known as the intrinsic viscosity, $k_{\mathrm{H}}$ is a constant known as the Huggins coefficient, and $\eta_{\mathrm{s}}$ is the viscosity of the solvent. The first, known as the Huggins equation, directly follows from rearranging this equation: $\frac{\eta - \eta_{\mathrm{s}}}{\eta_{\mathrm{s}} c} = [\eta] + k_{\mathrm{H}}\, [\eta]^2\, c$. The second, known as the Kraemer equation, follows from the approximation that $\ln\left(\eta/\eta_{\mathrm{s}}\right) \approx \left(\eta/\eta_{\mathrm{s}}\right) - 1 - \frac{1}{2}\left(\eta/\eta_{\mathrm{s}} - 1\right)^2$; substituting from the virial expansion for $\eta/\eta_{\mathrm{s}}$ then yields the Kraemer equation: $\frac{\ln(\eta/\eta_{\mathrm{s}})}{c} = [\eta] + \left(k_{\mathrm{H}} - \frac{1}{2}\right)[\eta]^2\, c$. **c** Fitting both Huggins and Kraemer equations to our data and extrapolating to $c = 0$ then yields a direct determination of the intrinsic viscosity; we then use the established relation $c^* = 0.77/\,[\eta]$ (9) to directly compute $c^*$, which renders a value of $0.05 \pm 0.03$ w/w% for PEO 5 MDa dissolved in BMB.

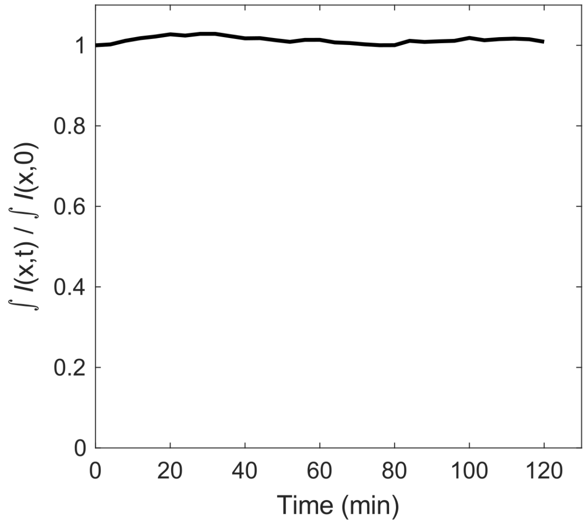


**Fig. S5. Minimal photobleaching of $O_2$ dye.** the integrated fluorescent signal measured for the liquid medium containing the $O_2$ probe does not decay over the experimental duration, directly demonstrating that photobleaching is minimal.

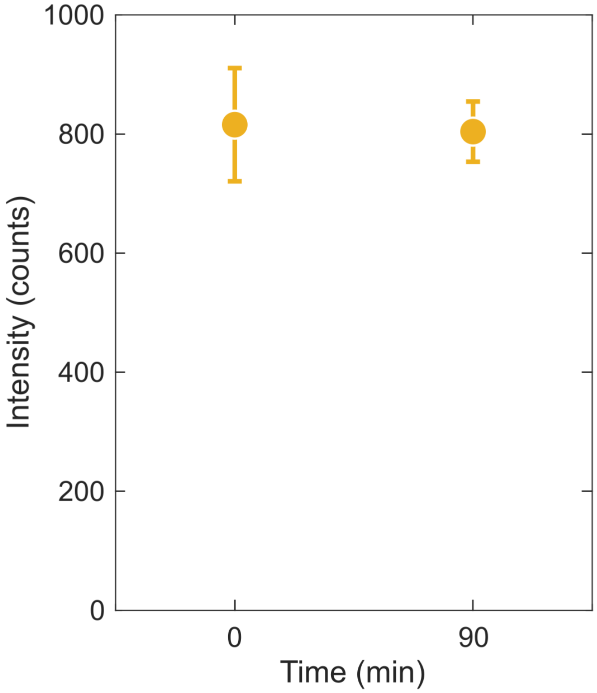


**Fig. S6. Minimal effect of bacterial chemical factors on $O_2$ dye.** We measure the fluorescence intensity of the supernatant in two identical bacterial suspensions (using identical imaging conditions, same cell concentration as in Fig. 1F of the main text) after incubation for either $1$ min or $90$ min. We observe no change in the fluorescence intensity, indicating that any biochemical changes to the composition of the liquid induced by bacterial activity also do not change the fluorescence intensity over the experimental duration, consistent with previous findings for *E. coli* (10).

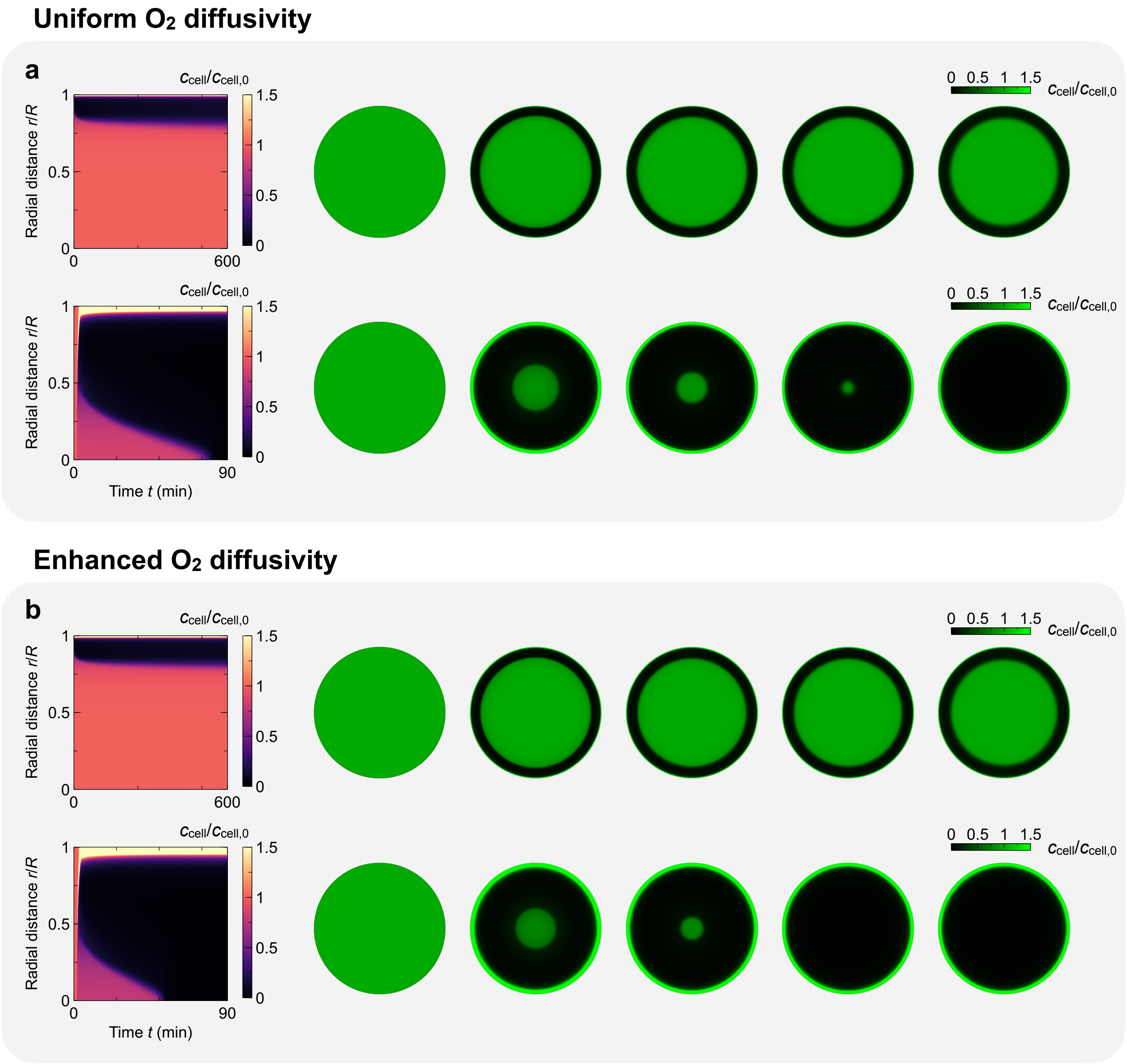


**Fig. S7.** Simulation results assuming uniform oxygen diffusivity (A) and $c_{\mathrm{cell}}$-dependent oxygen diffusivity (B). A includes the results that are also presented in Fig. 3E (top row) and 3G (bottom row) of the main text. In B, the top and bottom rows correspond to the same conditions as in A.

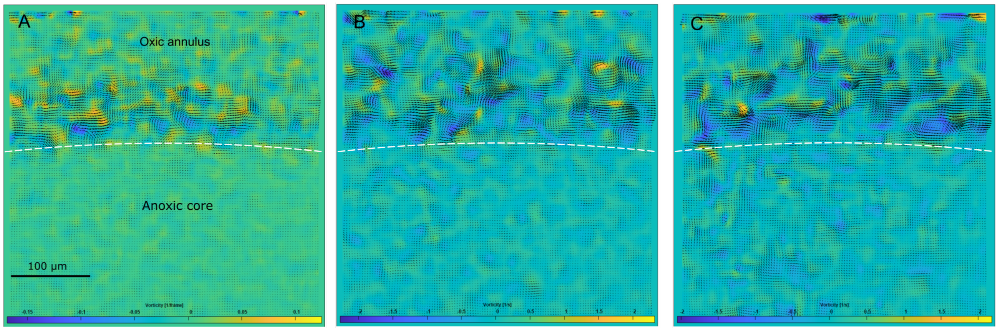


**Fig. S8. Flow field measurements at different depths in the droplet.** We used PIV to measure the flow field at different heights within the Hele-Shaw cell $h = 20, 30,$ and $40\ \mu\mathrm{m}$, presented in *A*, *B*, and *C*, respectively. Cell concentration is $c_{\mathrm{cell}} = 8 \times 10^{10} \mathrm{cell/mL}$. The depth is measured from the bottom surface. The images were acquired using confocal imaging with the optical section $\approx 3\ \mu\mathrm{m}$ thick.

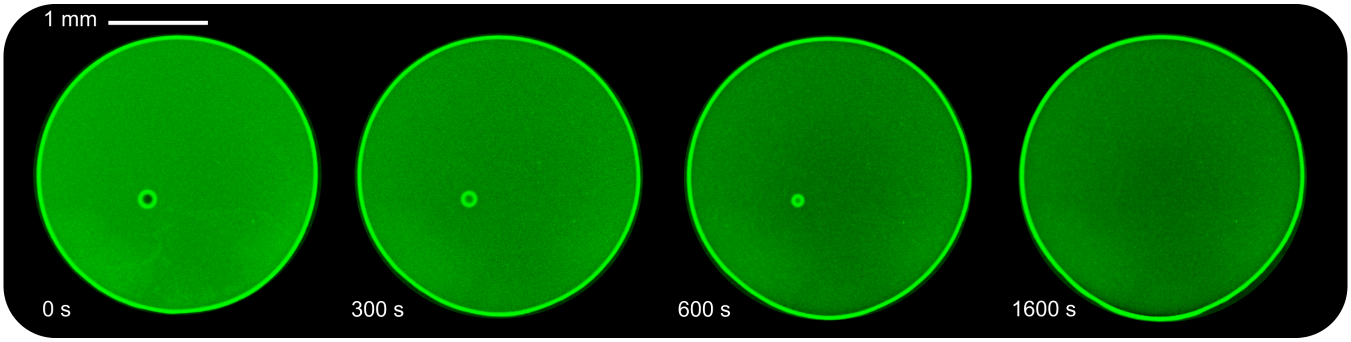


**Fig. S9. Dynamics of a nonmotile *E. coli* suspension in BMB.** In contrast to the motile cell suspension, due to the lack of motility, the nonmotile cells do not undergo phase separation. The cell concentration is $c_{\mathrm{cell}} = 4 \times 10^{10}\ \mathrm{cells/mL}$. Higher intensity corresponds to larger $c_{\mathrm{cell}}$. The hollow circle is an air bubble that gradually dissolves.

| Physical parameters | Definition | Range of values | Reference |
|---|---|---|---|
| $c_{\mathrm{cell},0}$ | Initial bacterial concentration | $5 \times 10^9 - 8 \times 10^{10}$ cells mL$^{-1}$ | Measured |
| $k_{\mathrm{O_2},0}$ | Bacterial maximal $O_2$ uptake rate | $4 \times 10^4 - 3 \times 10^5$ molecule (s cell)$^{-1}$ | Fitted |
| $D_{\mathrm{cell},0}$ | Diffusivity coefficient of motile bacteria | 220 $\mu$m$^2$ s$^{-1}$ | Measured |
| $\chi_0$ | Aerotactic sensitivity of motile bacteria | 2400 $\mu$m$^2$ s$^{-1}$ | Ref. (11) |
| $K$ | Michaelis-Menten constant | 1 $\mu$M | Ref. (12, 13) |
| $K_\chi$ | Aerotactic dissociation constant | 0.7 $\mu$M | Refs. (11, 14) |
| $D_{\mathrm{O_2}}$ | $O_2$ diffusivity coefficient | 2600 $\mu$m$^2$ s$^{-1}$ | Ref. (12, 15) |
| $c_{\mathrm{sat}}$ | $O_2$ saturation concentration | 0.274 mM | Refs. (12, 16) |
| $c_{\mathrm{crit}}$ | $O_2$ loss of motility concentration | 1 nM | Ref. (6, 14) |
| $k_{\mathrm{t}}$ | $O_2$ mass transfer coefficient | 0.005 mm s$^{-1}$ | Ref. (17) |
| $R$ | Drop radius | 1-3.5 mm | Measured |
| Characteristic scales (Low $k_{\mathrm{O_2},0}$) | | | |
| $\ell_{\mathrm{c}} = \ell_{\mathrm{O_2}} = [D_{\mathrm{O_2}} c_{\mathrm{sat}}/(k_{\mathrm{O_2}} c_{\mathrm{cell},0})]^{1/2}$ | $O_2$ penetration length | 366-1464 $\mu$m | |
| $t_{\mathrm{c}} = \ell_{\mathrm{O_2}}^2/\chi_0$ | Characteristic time scale | 0.9-15 min | |
| $c_{\mathrm{c}} = c_{\mathrm{sat}}$ | Characteristic $O_2$ concentration | 0.274 mM | |
| $c_{\mathrm{cell,c}} = c_{\mathrm{cell},0}$ | Characteristic bacterial concentration | $5 \times 10^9 - 8 \times 10^{10}$ cells mL$^{-1}$ | |
| Dimensionless parameters | | | |
| $\tilde{R} \equiv R/\ell_{\mathrm{c}}$ | Drop radius/$O_2$ penetration length | 0.1-10 | |
| $\tilde{k}_{\mathrm{t}} \equiv k_{\mathrm{t}} \ell_{\mathrm{c}}/D_{\mathrm{O_2}}$ | $O_2$interfacial transport rate/$O_2$ diffusive rate | 0.7-2.8 | |
| $\Gamma \equiv \chi_0/D_{\mathrm{O_2}}$ | Bacterial aerotaxis/$O_2$ diffusion | 0.92 | |
| $\tilde{D}_{\mathrm{cell},0} \equiv D_{\mathrm{cell},0}/\chi_0$ | Bacterial diffusion/Bacterial aerotaxis | 0.092 | |
| $(\tilde{K}, \tilde{K}_\chi, \tilde{c}_{\mathrm{crit}}) \equiv (K, K_\chi, c_{\mathrm{crit}})/c_{\mathrm{sat}}$ | Char. $O_2$ conc./$O_2$ saturation conc. | (3.6,2.6,0.011) $\times$ 10$^{-3}$ | |

**Table S2. Estimates and experimental measurements of the physical and dimensionless parameters used in the model.**
